# Complexation and Sequestration of BMP-2 from an ECM Mimetic Hyaluronan Gel for Improved Bone Formation 

**DOI:** 10.1371/journal.pone.0078551

**Published:** 2013-10-22

**Authors:** Marta Kisiel, Agnieszka S. Klar, Manuela Ventura, Jos Buijs, Marc-Krystelle Mafina, Simon M. Cool, Jöns Hilborn

**Affiliations:** 1 Division of Polymers Chemistry, Department of Chemistry-ångström, Science for Life Laboratory, Uppsala University, Uppsala, Sweden; 2 Tissue Biology Research Unit, Department of Surgery, University Children’s Hospital, Zurich, Switzerland; 3 Biomaterials, Radboud University Nijmegen Medical Centre, Nijmegen, Netherlands; 4 Science for Life Laboratory, GE Healthcare, Stockholm, Sweden; 5 School of Engineering and Materials Science, Queen Mary University of London, London, UK; 6 Glycotherapeutics Group, Institute of Medical Biology, A*STAR, Singapore, Singapore, Singapore; University of Patras, Greece

## Abstract

Bone morphogenetic protein-2 (BMP-2) is considered a promising adjuvant for the treatment of skeletal non-union and spinal fusion. However, BMP-2 delivery in a conventional collagen scaffold necessitates a high dose to achieve an efficacious outcome. To lower its effective dose, we precomplexed BMP-2 with the glycosaminoglycans (GAGs) dermatan sulfate (DS) or heparin (HP), prior to loading it into a hyaluronic acid (HA) hydrogel. *In vitro* release studies showed that BMP-2 precomplexed with DS or HP had a prolonged delivery compared to without GAG. BMP-2-DS complexes achieved a slightly faster release in the first 24 h than HP; however, both delivered BMP-2 for an equal duration. Analysis of the kinetic interaction between BMP-2 and DS or HP showed that HP had approximately 10 times higher affinity for BMP-2 than DS, yet it equally stabilized the protein, as determined by alkaline phosphatase activity. Ectopic bone formation assays at subcutaneous sites in rats demonstrated that HA hydrogel-delivered BMP-2 precomplexed with GAG induced twice the volume of bone compared with BMP-2 delivered uncomplexed to GAG.

## Introduction

Worldwide, patients continue to suffer from bone non-unions. Gold standard treatment relies on the continued use of autologous bone graft obtained from the patient’s own iliac crest [1]. This bone source has a limited quantity and the quality is dependent on the individual patient, which reduces its therapeutic potential [2]. Thus, bone repair by tissue engineering systems has attracted broad attention. Despite the continuing development of hormones and other bone-stimulating molecules, bone morphogenetic proteins (BMPs) remain the most potent inducers of bone formation *in vivo* [3]. In particular, BMP-2 is widely recognized to be one of the most powerful osteoinductive factors for bone regeneration [4,5] and was originally identified as a factor in bone tissue that in extracted form could stimulate bone formation when added exogenously to an extraosseous site [6]. Moreover, human recombinant BMP-2 [7], has proven to be highly efficient as a bone-inducing adjuvant in animals. 

Endogenous BMP-2 is also important for normal bone homeostasis and is upregulated immediately following bone trauma [8] and actively contributes to the recruitment, proliferation and differentiation of osteoprogenitor cells during the bone healing process [9]. In the clinical setting, BMP-2 absorbed into a bovine collagen type I sponge has proven to be effective in the treatment of degenerative disc disease (spinal fusion) and fracture non-union [10,11]. However, excessive dosing has been associated with adverse events that include tissue edema and ossification at undesired sites [12,13]. There is also concern because the systemic half-life of BMP-2 is short and FDA-approved delivery is reliant on a collagen sponge with low affinity for BMP-2 [14], so requiring supra-physiological doses in order to achieve an efficacious outcome [15]. 

Recent evidence by our group and others [16,17] suggests that BMP-2-induced bone formation is largely dependent on stability of BMP-2 and its release kinetics, with a controlled release enhancing the effect. Long-term BMP-2 delivery increases bone-healing rates compared with short-term delivery at an equal dose [18,19]. As a consequence, a number of delivery strategies aimed at improving BMP-2 dose-effectiveness have been developed. Our group, along with others, has shown that hyaluronic acid (HA) hydrogels are suitable for bone tissue engineering applications [20-23]. HA is a natural extracellular matrix glycosaminoglycan (GAG) that regulates several biological processes, including cell migration, proliferation, differentiation and wound healing [24]. *In vivo*, HA is degraded by the action of oxygen free radicals [25] and hyaluronidases [26,27]. Also, HA hydrogels are non-immunogenic and have been successfully utilized as scaffolds for BMP-2 delivery both in preclinical [20,28,29] and clinical [13] trials. As an injectable device, HA permits *in situ* administration in a minimally invasive manner [21,30]. Although promising characteristics, HA hydrogels share a problem with many similar materials, namely insufficient control of BMP-2 release. This is because many hydrogels rapidly releases BMP-2 through a passive diffusion mechanism [28]. Although BMP-2 could be covalently linked to this polymeric scaffold [31] such a chemical modification may compromise BMP-2 activity. Also, electrostatic immobilization of BMP-2 on a basement membrane proteoglycan (perlecan domain I) covalently conjugated to a HA hydrogel has been attempted [32]. However this strategy whilst sustaining the release of active BMP-2, is limited by the elaborated multi-step bioconjugation.

In the present study we aim to optimize the delivery of BMP-2 from an HA hydrogel through the simple addition of a natural extracellular matrix (ECM) glycosaminoglycan (GAG). Previous reports have shown that the incorporation of GAGs, such as heparin (HP), in a polymer carrier significantly improves BMP-2-mediated bone formation [33,34]. Surprisingly, little information has been published regarding the role that other GAGs play in mediating BMP-2 activity, although this is rapidly gaining interest among researchers. Dermatan sulfate (DS), also known as chondroitin sulfate B, has be shown to interact with and mediate the activity of HGF [35], FGF-2 and PDGF [36]. However, to date little is known of a role for DS in BMP-2-induced osteogenesis. To address this, we investigated how DS influences BMP-2 activity compared with HP. We then went on to assess whether DS improved the *in vivo* bone forming capacity of BMP-2. Our strategy was to pre-complex BMP-2 with either DS or HP prior to loading it into the HA hydrogel and to compare the *in vitro* release of BMP-2. Next, we characterized the interactions between BMP-2 and DS or HP by surface plasma resonance (SPR) to study non-covalent interactions and to measure the affinity constant. Then, using an alkaline phosphatase activity assay, we determined whether the BMP-2 released from the constructs was bioactive. Finally, we assessed gel constructs in a subcutaneous rat model for ectopic bone formation using micro-CT, scanning electron microscopy (SEM), histology and immunohistochemistry (IHC). 

## Results

### 1: Construct preparation

While preparing the constructs for bone engineering, we first precomplexed BMP-2 with DS or HP at a 1:5 ratio and then loaded the complex into the HA hydrogel scaffold (Figure S1). The gel was formed within 1 min by mixing all components in a dual syringe system. The mixing triggered a spontaneous cross-linking reaction of the two polymers with complementary reactive functionalities under neutral aqueous conditions. The advantage of BMP-2 precomplexation with heparin has been demonstrated in a scaffold loaded with BMP-2. In a study by Johnson et al., BMP-2 precomplexed with HP prior to adding a collagen carrier yielded 20% more bone formation in a femoral defect model compared with BMP-2 adsorbed into collagen with incorporated HP [37][. Bramono et al. found that a BMP-2:HP ratio of 1:5 was the most favorable for enhancing ectopic bone formation [38]; therefore, we used this proportion in our study as well. 

#### 2: In vitro BMP-2 release kinetics and bioactivity

**Figure 1:**
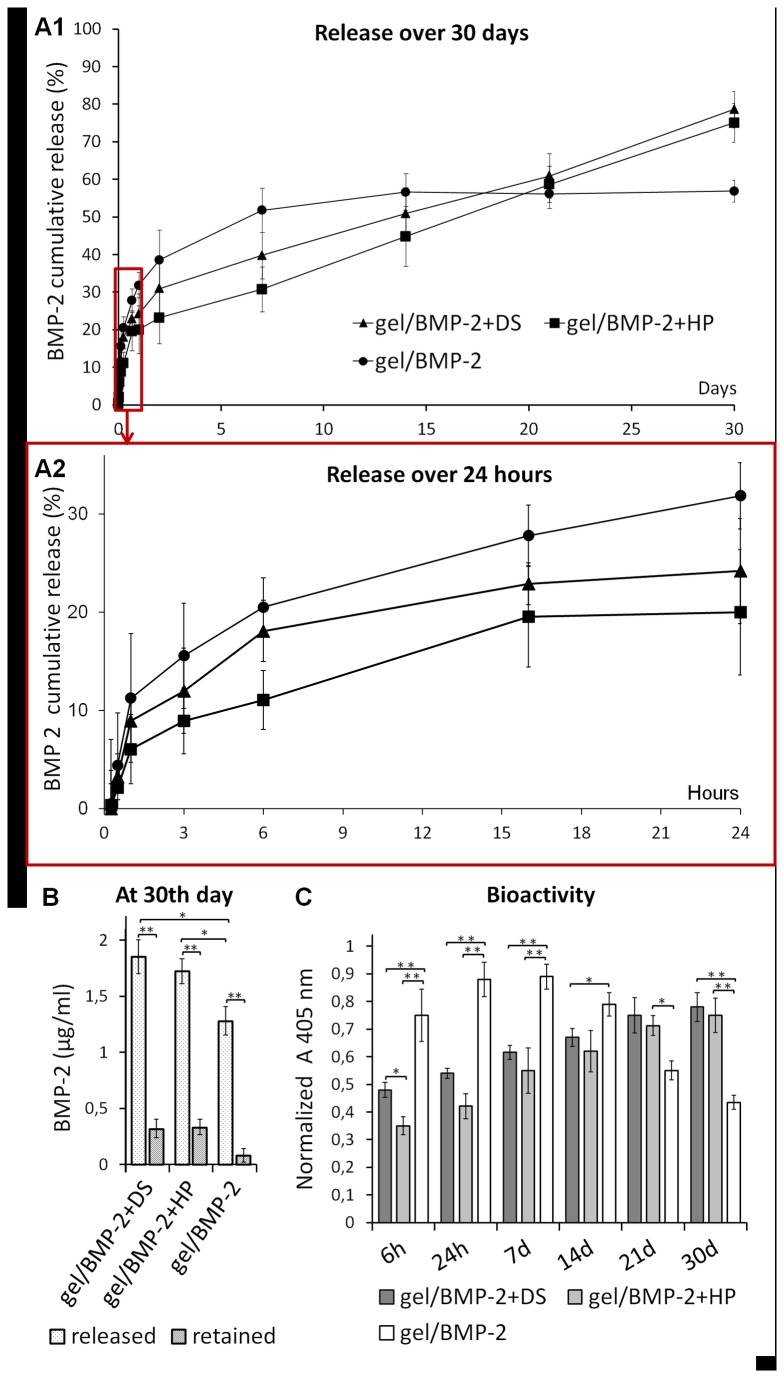
*In* *vitro* release of BMP-2 from gel/BMP-2+DS, gel/BMP-2+HP and gel/BMP-2 over 30 days (A1) and over 24 h (A2). The amount of BMP-2 measured by ELISA is shown as the cumulative release normalized to the control release. (B) BMP-2 released/retained from the constructs after 30 days. (C) The bioactivity of BMP-2 released over 30 days as assessed by the ALP activity of myoblast cells. The values represent the mean ± SD (n=3). Error bars represent the SD (n=3); *p<0.05 and **p<0.01.

To evaluate the extent to which BMP-2 premixed with DS or HP could delay the release of BMP-2 from the HA hydrogel through affinity binding interactions, we performed an *in vitro* release study over 30 days. The amount of BMP-2 released from all constructs was first determined using an ELISA, and the biological activity of the constructs was examined via the ALP assay (Figure 1). We are aware that the protein is sensitive and could easily absorb to the vial surface and form aggregates in the release medium [39]. Therefore, to avoid erroneous results that could be due to the dilution of BMP-2, at each time point, the result was compared with the result obtained from the incubation of free BMP-2 at an adequate concentration (control). Furthermore, we measured the amount of protein that was retained in the constructs after 30 days (Figure 1B). The cumulative release consisted of two phases: an initial release lasting up to 24 h (Figure 1A2) and a subsequent, slower release lasting up to the 30^th^ day (Figure 1A1). There was a clear difference in the initial release profile between the constructs. The most rapid release was observed in the HA hydrogel loaded with only BMP-2. From 6h up to 24h we observed significant difference in the amount of BMP-2 released from gel/BMP-2 with DS compared to gel with only BMP-2 (p<0,05). The HA hydrogels with BMP-2 premixed with DS exhibited a larger initial release compared with the construct containing HP. However, the further release of BMP-2 from gel/BMP-2+DS and gel/BMP-2+HP remained relatively constant and sustained over 30 days. The total final release was 79% for DS and 75% for HP and was significantly higher than the total final release from gel/BMP-2. In contrast; the release from gel/BMP-2 reached a plateau after 7 days at 56% of the total release and did not increase much before the end of the experiment. 

To determine the amount of BMP-2 retained in the constructs after 30 days, we digested constructs with a hyaluronidase solution. We found that not all BMP-2 was released from the scaffolds by day 30 (Figure 1B). Approximately 15% of the initial BMP-2 remained in both constructs loaded with precomplexed BMP-2, while approximately 5% was found in gel/BMP-2. Summing released and retained BMP-2 we found that the part of BMP-2 loaded in the gels was lost. The amount of retained BMP-2 was significantly higher in gel/BMP-2+DS or HP compared to gel/BMP-2 (p<0.05). It might be due to aggregation or adhesion to the storage tube surface upon release experiment [20]. As expected, no BMP-2 was detected from the HA hydrogels that were not loaded with BMP-2 (negative controls). Our observations of the release profile of BMP-2 alone in the HA hydrogel were similar to those of our previous studies. Accordingly, there was an initial burst release of BMP-2 in the first 24 h and then almost a complete release during the first week [39]. 

**Figure 2:**
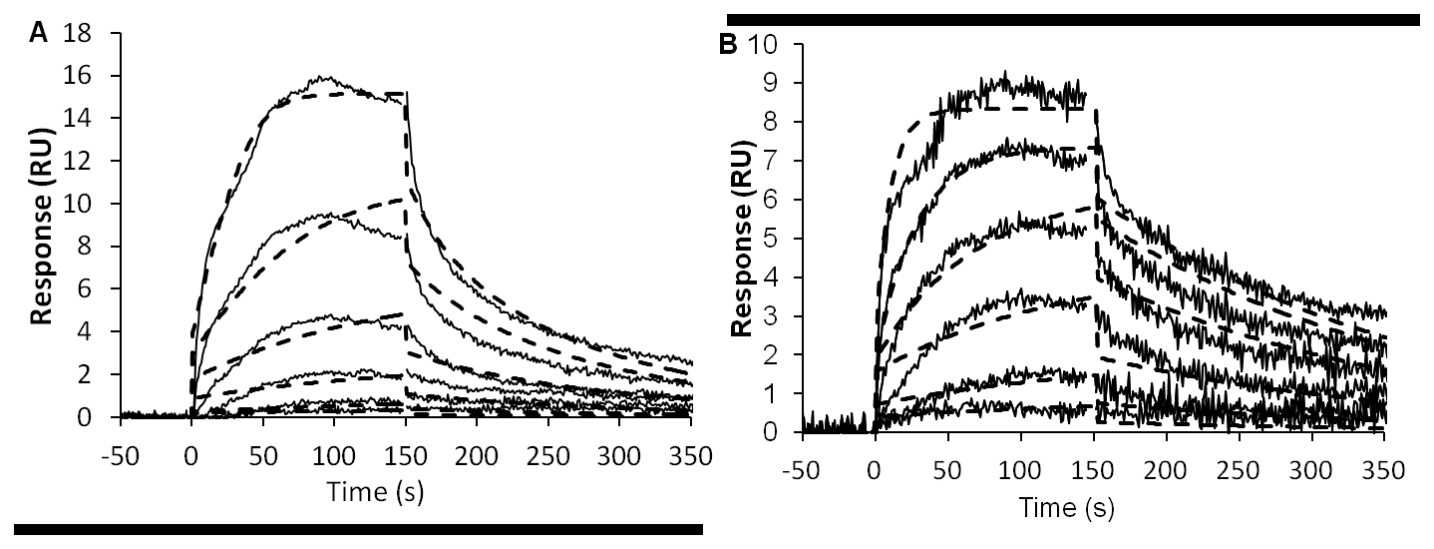
Kinetic analyses of BMP-2 binding to (A) dermatan sulfate (DS) and (B) heparin (HP). Solid lines represent the binding curves for six BMP-2 concentrations ranging from 0.2 to 50 nM. The dotted lines depict the result of a global fit of a 1:1 interaction model to the binding data. The binding affinity, K_d_, was calculated as the ratio of the dissociation and association rate constants, k_d_/k_a_. The study yielded an affinity constant, K_d_, of 2.0±0.8 x 10^-8^ M for the binding of BMP-2 to DS and a K_d_ of 2.4±0.3 x10^-9^ M for the binding to HP. The standard deviation is based on the variation between duplicate experiments.

We then studied the biological activity of BMP-2 released from constructs using ALP assays with the C2C12 cell line. In the ALP activity assay only bioactive BMP-2 is able to trigger differentiation of bone cells. The ALP activity of C2C12 cells did not increase in the control supernatant obtained from the HA hydrogel without BMP-2, and this result mirrored the result obtained for cells cultured in media alone (negative control). The addition of fresh BMP-2 to the culture medium enhanced ALP activity (positive control). The ALP activity resulting from the BMP-2 released from all constructs at each time point over 30 days is shown in Figure 2C. The HA hydrogel with precomplexed BMP-2 demonstrated low ALP activity during the 1^st^ week, followed by an increase during the 3^rd^ and 4^th^ weeks. In contrast, the HA hydrogel without premixed BMP-2 exhibited increased ALP activity during the first week that was reduced during the 3^rd^ and 4^th^ weeks of the study. 

### 3: BMP-2 binds to HP and DS

To understand the mechanism of BMP-2 release from the gel construct, we examined the interactions between BMP-2 and DS or HP at the molecular level using surface plasmon resonance (Figure 2). A kinetic analysis of those interactions was performed by injecting a concentration series of BMP-2 over immobilized DS (Figure 2A) or HP (Figure 2B). The binding affinity, Kd, was calculated as the ratio of the dissociation and association rate constants, k_d_/k_a_. The kinetic data were fitted by the 1:1 Langmuir model. The interaction with BMP-2 yielded an affinity constant, Kd, of 2±0.8 x 10^-8^ M for the binding to DS and a Kd of 2.4±0.3 x10^-9^ M for the binding to HP. An approximately three-fold slower association and a three-fold faster dissociation explained the lower binding affinity of BMP-2 for DS compared with that for HP.

### 4: In vivo bone efficacy

#### 4.1: Bone volume analysis

**Figure 3:**
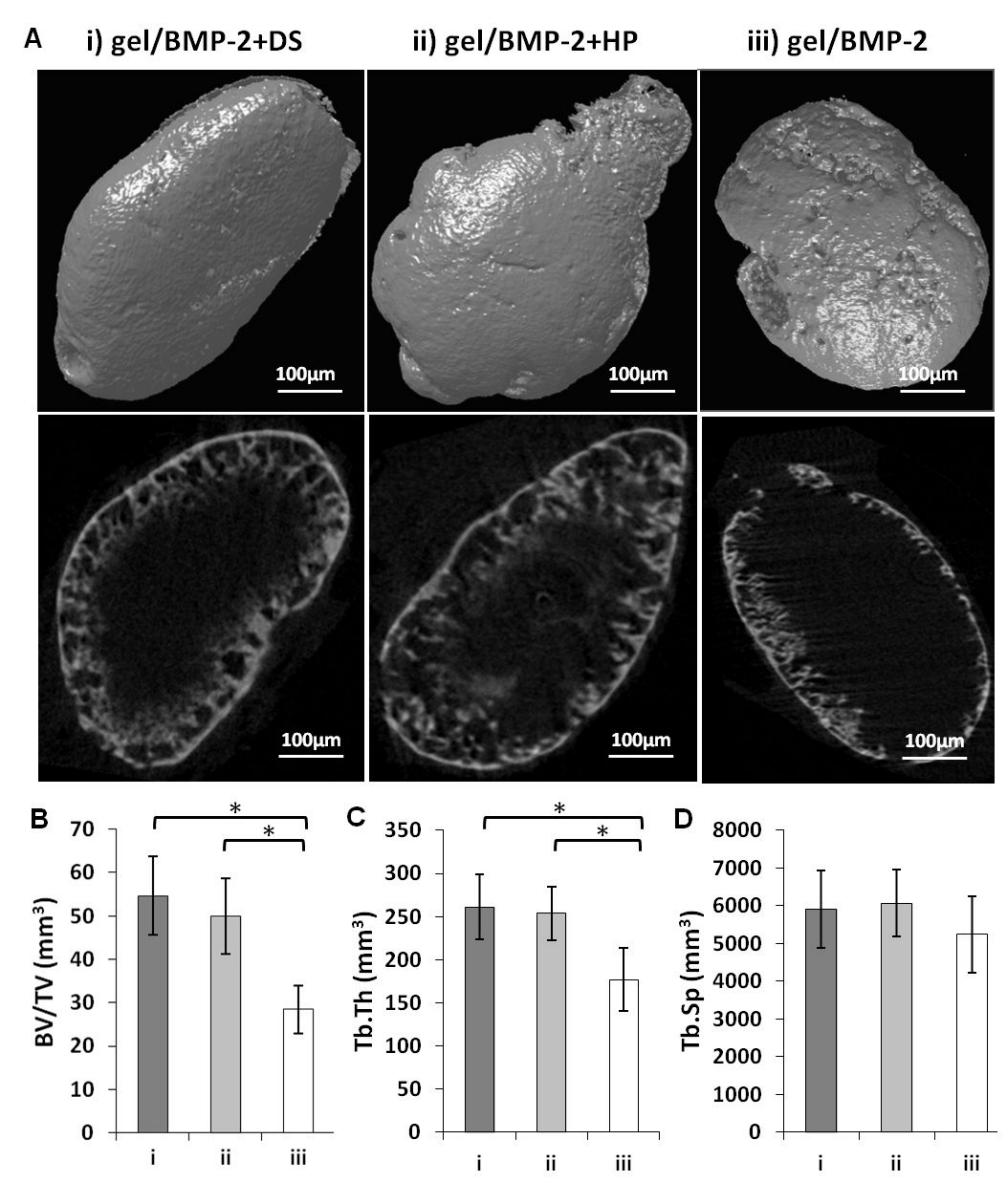
Micro-CT analysis. (A) 3D (upper row) and 2D (lower row) image of the surface of ectopic bones formed 6 weeks post-injection of (i) gel/BMP-2+DS, (ii) gel/BMP-2+HP and (iii) gel/ BMP-2. (B) The average bone volume/tissue volume (BV/TV) ratio, (C) the average trabecular thickness (Tb.Th.) and the average trabecular separation (Tb. Sp.) were calculated, and the values represent the mean ± SD for n=6; *p<0.05.

To determine the *in vivo* efficacy of the constructs, the HA hydrogel containing BMP-2 precomplexed with DS or HP, containing BMP-2 only, or without the protein was injected at subcutaneous sites in rats. We injected a 200 µL volume of constructs containing HA hydrogel with 4 µg of BMP-2 with or without 20 µg of HP or DS. We used HA hydrogel alone as a negative control. The constructs were prepared as described in section 3.1 (Figure S1). No complications occurred after the surgical intervention, and the animals remained in good health during the entire study. After 6 weeks, rats were sacrificed, and ectopic bone was harvested. All scaffolds containing BMP-2 induced bone, while no evidence of ectopic bone formation or remaining gel was detected in the groups injected with scaffolds lacking protein. HA degradation did not result in any signs of an inflammatory reaction or an influx of macrophages. The ectopically formed bone tissue was rounded in shape and firmly attached to the surrounding tissue. The ectopic bone tissues were harvested, fixed and subjected to micro-CT analysis using a pre-determined threshold of cortical bone for direct clinical translation. The 3D and 2D reconstruction of a representative sample from each group is shown in Figure 3A i-iii. Micro-CT showed that BMP-2 precomplexed with DS or HP significantly improved the potential of BMP-2 to induce bone formation compared with the protein alone when the same amount was delivered via the HA hydrogel. The quantitative analysis revealed that the average volume of ectopic bone formed at the site of the construct with GAGs was significantly higher (p<0.5) than the volume of bone induced by the HA hydrogel with only BMP-2 (Figure 3B). The volumes of ectopic bone formed by gel/BMP-2+DS and gel/BMP-2+HP were 55 and 52 mm^3^, respectively, and were two-fold higher (p<0.05) than the volume of bone generated by gel/BMP, which was 28 mm^3^. Furthermore, the mean trabecular thickness (Tb.Th.) was significantly larger in ectopic bones formed by gel/BMP+DS or HP compared to those induced by gel with only BMP-2 (Figure 3 C). However, the mean trabecular separation (Tb.Sp.) was only slightly greater in ectopic bone areas formed by gel containing BMP-2 and DS or HP than those induced by gel with BMP-2 alone (Figure 3 D). 

#### 4.2: Bone structure

**Figure 4:**
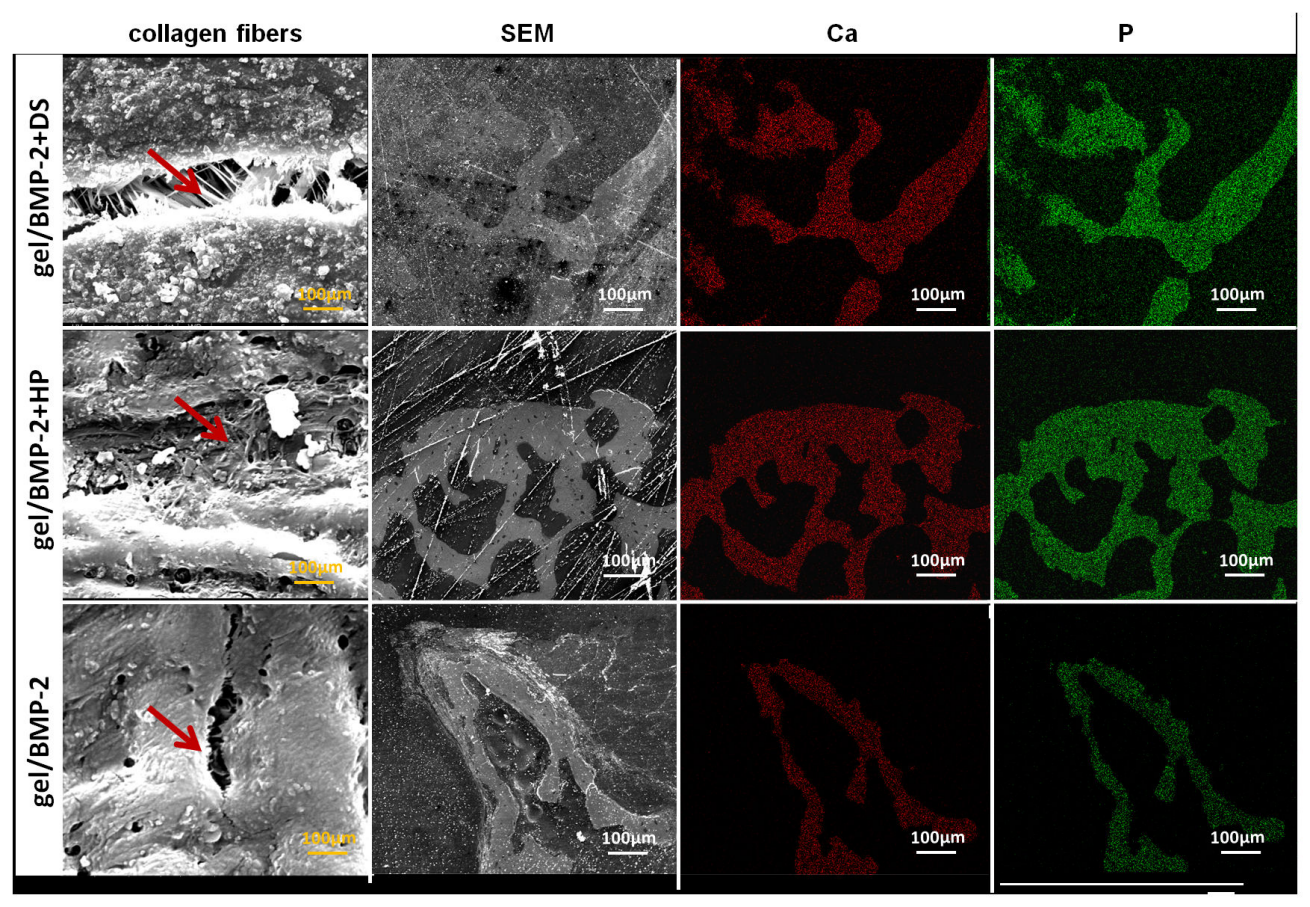
Six weeks following implantation, the ectopic bone formed by gel/BMP-2+DS, gel/BMP-2+HP and gel/BMP-2 was evaluated by histology via Masson’s Ttrichrome staining and osteocalcin (OC) immunostaining. B indicates a trabecular bone structure; nuclei were stained with DAPI.

The results of the micro-CT analysis were further verified with standard histology and immunohistochemistry (Figure 4). The same as 2D sections from micro-CT (Figure 3A, lower row), the transverse sections of ectopic bone stained with Masson’s Trichrome showed that the bone was formed at the edges rather than in the center of the sample. The central part of the sample was not filled with bone tissue and the transversal sections appear as a ring. The surface of bone area was, however, remarkably larger in ectopic bones formed in place of the HA gel/BMP-2+DS or HP comparing to the HA gel/BMP-2. The sections of ectopic bones induced by BMP-2 premixed with GAGs exhibited a dense trabecular structure interwoven with cellular elements than BMP-2 not complexed. There was no sign of fibrotic tissue in any area of the ectopic bone. 

Furthermore, we analyzed osteocalcin (OC) expression in the ectopic bone sections (Figure 4, right panel). OC is a marker of bone formation and ultimately a proof that the tissue formed is bone. The equal signal was showed in all specimens at the areas where the bone was previously shown by Masson’s Trichrome staining. In all groups, we showed that OC was positively stained not only in osteoblasts, but also in their surrounding matrix (Figure 4, right panel, inserts). As a positive control we used a native rat cranium tissue (Figure S2).

#### 4.3: Bone mineral composition

**Figure 5:**
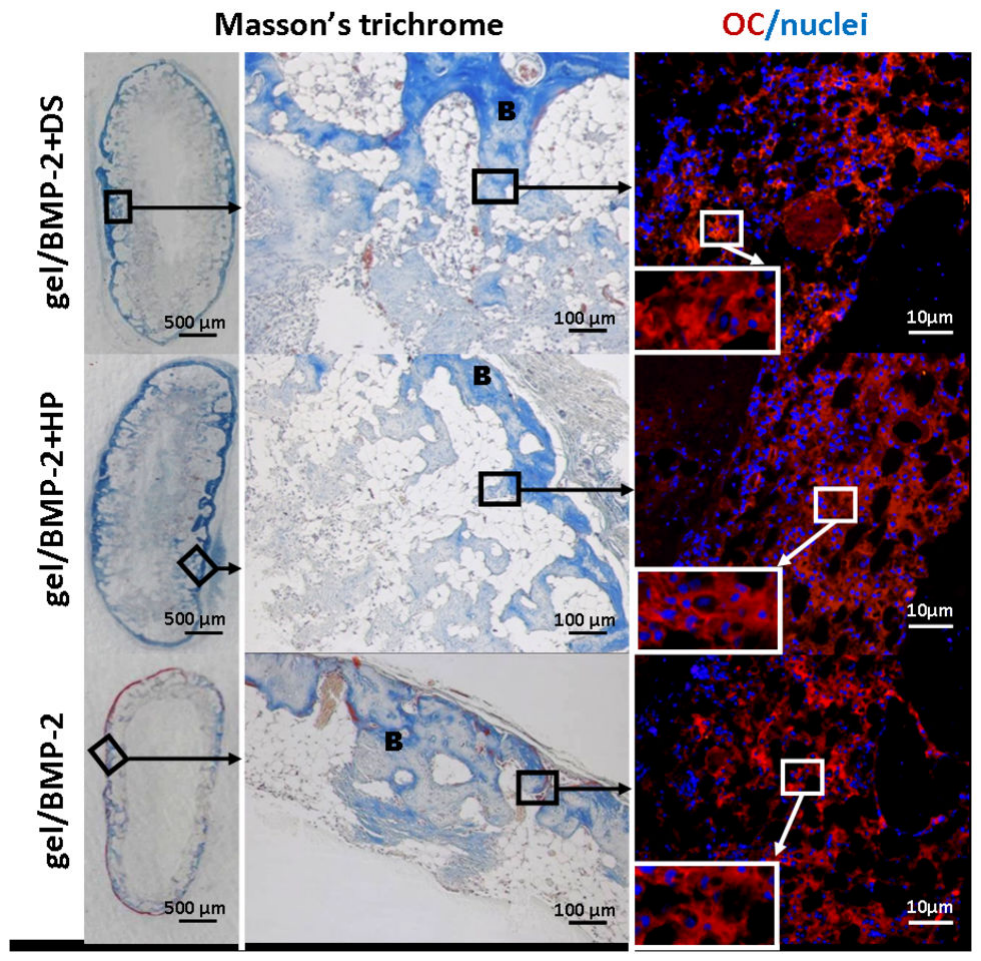
Elemental mapping of the surface of ectopic bone induced by gel/BMP-2+DS, gel/BMP-2+HP and gel/ BMP-2. Collagen fibers, secondary electron (SEM), calcium (Ca) and phosphorus (P) are shown. SEM was performed via gold coating.

We then examined ectopic bone samples by SEM. We showed that ectopic bone with more interconnected collagen fibers areas was formed from the gel/BMP-2+DS and gel/BMP-2+HP compared to the gel/BMP-2 (Figure 5). Furthermore, mapping analysis through SEM allowed the bone samples to be viewed in terms of their elemental distribution, with calcium (Ca) and phosphorus (P) . Backscattered electron mapping on ectopically formed bone for the three groups illustrated increased distribution of lighter and darker gray regions. This heterogeneity arises from larger distributions of higher and lower atomic number elements in the bone of the gel/BMP-2 (Figure 5). These results were confirmed by point analyses using elemental mapping of relative intensity of Ca and P, in which all groups demonstrated more Ca and P in lighter areas compared to corresponding darker areas.

#### 4.4: Angiogenesis

**Figure 6:**
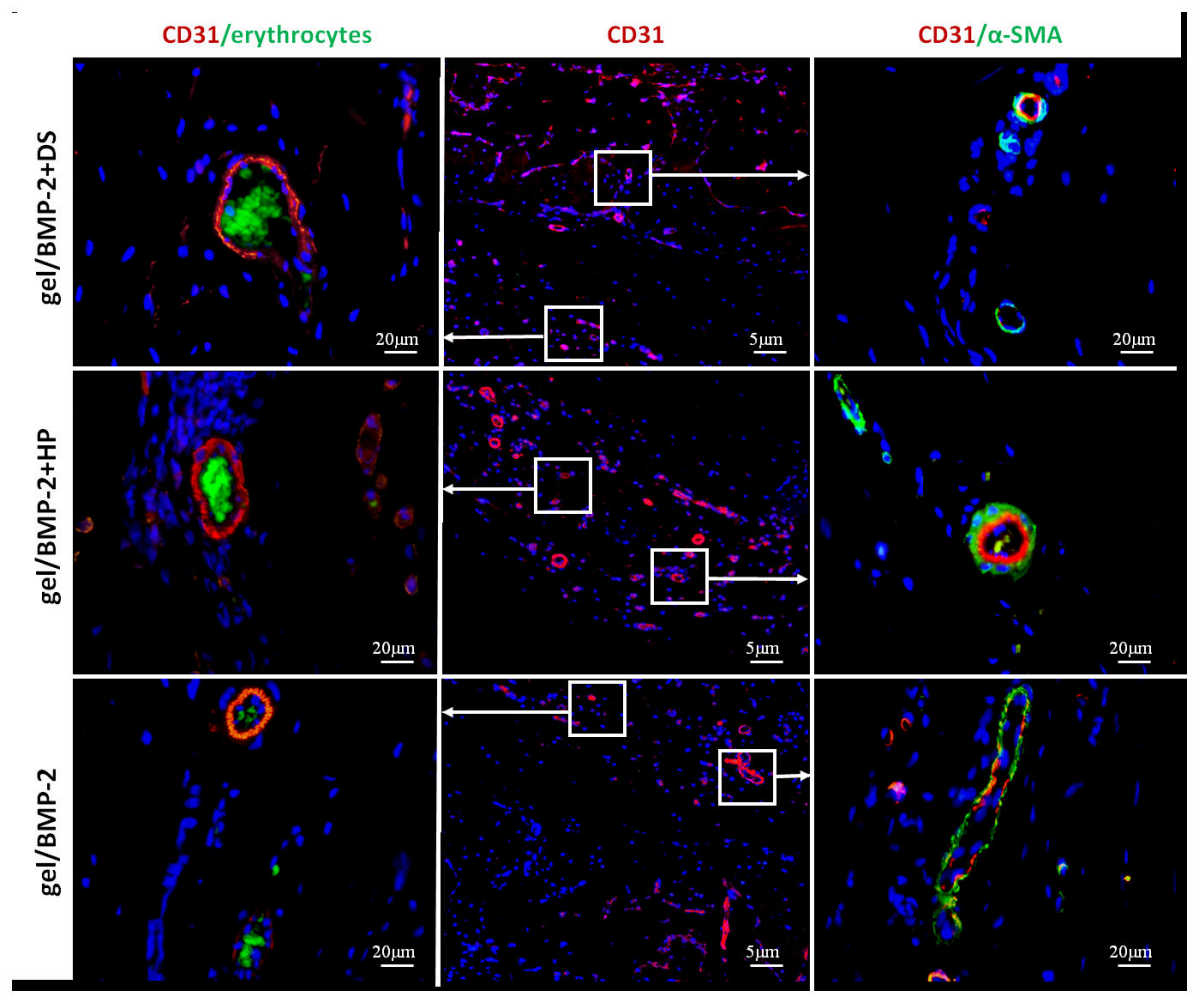
The cross sections of ectopic bone formed due to gel/BMP-2+DS, gel/BMP-2+HP and gel/BMP-2 were immunostained with CD31 (red) and α-SMA (green in right panel). The erythrocytes were visualized by green autofluorescence (green in left panel). The area showed in middle panel corresponds to area indicated in Figure 5. Cells nuclei were stained with DAPI (blue). The images are shown merged.

Because osteogenesis is dependent on blood vessel formation and bone is more likely deposited near blood vessels [40], we evaluated blood vessel formation in the ectopic bone after 6 weeks. To confirm that the observed vessels were maturated, sections were double-labeled with antibodies directed against the endothelial marker CD31 and mural cell marker α-SMA. As shown in Figure 4, osteoid in all groups was formed in the outer ring, which is where we detected the blood vessels. We observed that CD31-positive rat blood vessels (red) were penetrating the bone marrow of newly formed bone (Figure 6). Moreover, autofluorescent erythrocytes (green) were present in the lumen of all identified vessels. The autofluorescence of erythrocytes is due to hemoglobin content [41]. We also detected the presence of an outer layer of α-SMA-positive cells surrounding the CD31-positive vessels. Positive fluorescence could not be detected in sections exposed to the secondary antibody alone (data not shown). Native rat cranium tissue was used as a positive control (Figure S2). The CD31 expression patterns were equally distributed in all three groups. Taken together, these data demonstrate the presence of efficient host angiogenesis upon transplantation of all gel constructs.

## Discussion

BMP-2 plays an important role in the early stages of bone regeneration by recruiting local sources of skeletal progenitors and by determining their fate towards the osteogenic lineage. Current methods for therapeutic administration of BMP-2 for bone repair are associated with unacceptable side effects that have limited its therapeutic application. For instance, a clinical trial of alveolar cleft palates in children was prematurely ended due to a significant amount of postoperative swelling [12,13]. Moreover, patients treated with BMP-2 for posterolateral spinal fusion were found, during follow-up, to have an increased risk of cancer [25]. These issues arise due to the high concentration of the protein required for bone formation that is a result of the poor stability and short half-life of the protein *in vivo*. Molecules, such as BMP-2, that are sensitive and unstable require a delivery system that is able to protect the protein against premature degradation, regulate their *in vivo* release and provide delivery to the lesion site. We achieved such a delivery system in the current study using GAGs, such as dermatan sulfate (DS) and heparin (HP), in a polymer carrier system that controls the release of active BMP-2 to significantly enhance bone formation. 

Mature BMP-2 is a homodimer that has one heparin binding site in each of the N-terminal sequences [42,43]. The heparin binding site consists of several positively charged residues that are able to interact with ECM components, providing a protein gradient by restricting the diffusion of free BMP-2 [42]. HP is a highly sulfated GAG that is known to bind to BMP-2 [33] and stimulate its activity by protecting it from enzymatic degradation and the antagonistic actions of noggin [9,34,44]. In contrast, some *in vitro* studies indicate that HP may inhibit osteogenic activity by sequestering BMP-2 on the cell surface and mediating protein internalization [45]. Animal studies demonstrated that the incorporation of HP into various scaffolds [33,34], including HA hydrogels [46], enhanced bone formation. These results only confirm that HP has a multifunctional regulatory role, and the range of early osteogenic markers may not resemble favorable outcomes of *in situ* bone formation [38]. The long-term administration of HP increases the risk of developing osteoporosis, suggesting that a large dose of HP may not be appropriate for frequent systemic administrations [47,48]. On the other hand, DS was recently demonstrated to stimulate progenitor differentiation of osteoblasts, mediated by BMP-2 [49]. However, its role in BMP-2-mediated bone formation has not yet been elucidated [50]. 

To increase the possibility of direct molecular interactions, we precomplexed BMP-2 with DS or HP prior to loading into the HA hydrogel scaffold. Our 30-day *in vitro* release study showed that the presence of DS or HP significantly prolonged BMP-2 retention while non-complexed BMP-2 released more rapidly from the gel. The total BMP-2 amount released from gel/BMP-2+DS and gel/BMP-2+HP was quite similar. Furthermore, the amount of BMP-2 released from the constructs determined by ELISA corresponds to the results of ALP assay with C2C12 cells. This outcome confirmed that BMP-2 released from the constructs maintained its biological activity. 

Interestingly, the only significant difference in BMP-2 release from gel/BMP-2+DS and gel/BMP-2+HP was found in the first 24 h of the *in vitro* cumulative release experiment. Specifically, the HA hydrogel with BMP-2 precomplexed with DS exhibited a slightly faster release than that precomplexed with HP. We therefore performed kinetic SPR analysis that showed that the BMP-2-DS complex has an affinity slightly less than 10 times that of the BMP-2-HP complex. The demonstrated increased binding affinity between BMP-2 and HP compared with that between BMP-2 and DS may be due to the presence of more sulfate groups in HP. HP contains a sulfated disaccharide unit that is able to electrostatically bind to BMP-2 and prolong the protein stability [9]. Negatively charged sulfate groups in GAGs bind to positively charged amino groups in different GFs, including BMP-2 [51]. It was shown that the ability of sulfated GAGs to stimulate BMP-2 depends on the size and number of their sulfated groups [38]. The affinity of DS for BMP-2 has not been defined, although previous studies hypothesized that DS, like HP, binds to the protein. The possible mechanisms of DS interactions with BMP-2 are not clear but are likely similar to those of HP interactions (i.e., electrostatic interactions). Previous studies reported that DS regulates the activity of FGF-10 and FGF-7 and that the effect is dependent on sulfated groups and IdoA disaccharides [52]. However, the SPR assay may detect all possible bonding mechanisms, and according to some previous reports, the interactions between GAGs and BMP-2 are complex and may also involve non-sulfated saccharides [53].

Although the SPR assay demonstrated that DS binds to BMP-2 with a slightly lower affinity than the binding of HP to BMP-2, further *in vivo* evaluation showed that both binding pairs were physiologically relevant. Micro-CT analysis in ectopic bone models demonstrated that bone formation was equal for both the DS- and HP-BMP-2 precomplexed groups with bone volumes two folds larger than treatment with BMP-2 alone. In 2D images from micro-CT (Figure 3A (lower row)) and transversal sections stained with Masson’s Trichrome (Figure 4) we noted that bone formed in the ectopic models as oval rings with bone concentrated at the edges with voids in the central part of specimen. The remarkable difference was that ectopic bone formed with treatments of gel/BMP-2+DS or HP had thicker bone area that penetrated deeper in the middle of the specimen in contrast to bones induced by gel/BMP-2 alone (Figure 4). The evaluation of trabecular thickness and trabecular separation showed that the trabecular bone structure of the ectopic bone tissue had higher density in the samples where BMP-2 was precomplexed. The advantage of more organized trabecular network in bone is apparent since the spontaneous fractures in osteoporosis occurred due to weak continuity and connectivity of bone trabeculae that disrupt force transmission [54]. No areas in the histological sections demonstrated any unidentified regions i.e. possible undegraded gel. Furthermore, no giant cells were seen. This corroborates with earlier studies in our laboratory that showed complete degradation within 4 weeks [28]. Next we assayed for osteocalcin, an early marker of bone formation, at the sites where bone tissue was observed (Figure 4). Furthermore, as another indication of the quality of ectopic bones we performed SEM analysis. We observed that the collagen fibers in groups of gel/BMP-2-DS or HP were more interconnected (Figure 5). Then the ionic mapping analysis revealed that the ectopic bones formed by BMP-2 precomplexed with GAGs contained less calcium (Ca) and phosphor (P) compared to bones created by BMP-2 not complexed (Figure 6). Both ions indicate greater content of the mineralized matrix that is a structural bone component. In addition Ca is crucial for a signal transduction for bone cell responses [55]. As newly formed layers of bone contained lower counts of Ca and P compared to the older layers of bone, it can be concluded that gel/BMP-2 produces bone early on that has mineralized to higher extent than the bone formed by precomplex BMP-2 in gel. Also, the wider distribution in the grey intensity of back-scattered electrons from energy-dispersive spectrometry (EDS) [56] in samples with gel and precomplexed BMP-2 suggest an indication of the possible the presence of both older and more newly formed bone. The elemental mapping showing lower Ca/P intensity ratio for gel/BMP-2+DS than for gel/BMP-2+HP may be indicative of a difference in the mechanisms involved in the bone mineralisation process. Finally we addressed the presence of angiogenesis within the ectopic bones since vascularization is critical for bone formation [40]. In all groups the marrow space of bone was rich with blood vessels with CD31 positive endothelial cells surrounded by α-SMA positive myocytes (Figure 6). In addition the blood vessels contained erythrocytes in the lumen that clearly indicated their functionality (Figure 6). 

The evaluation of ectopic bones engineered by the HA hydrogel/BMP-2 with or without GAGs suggested that the bone formation mechanism by those constructs has much in common with the long bone healing after fracture. The woven bone begins forming on the hematoma edges and remodel subsequently in lamellar bone [57]. The ectopic bones at 6^th^ week post injection might be the result of following sequences: (i) progenitor cells are attracted to the gel releasing BMP-2 and differentiate into osteoblasts cells; (ii) providing an adequate supply of active BMP-2, cells are attracted and migrate toward the center of the scaffold and produce matrix of collagen and non-collagenous proteins such as osteocalcin and blood vessels; (iii) woven bone started mineralization. In human body complete bone regeneration after injury takes some years and although the bone formation in rats is more rapid further monitoring of ectopic bone formation would not give more useful information. Already the formation of hard callus allows for patients immobilization that, in turn, give an appropriate biomechanical stimuli for final bone remodeling [58]. 

In this study we showed a simple and efficient system that significantly enhances BMP-2 induced bone repair. We attributed this outcome to molecular interactions occurring between active molecules in the gel environment. In Figure S3 we suggested the mechanism of the protein release from gel/BMP-2+DS or HP and gel/BMP-2. DS, like HP, binds to BMP-2 and the size of the complex might directly slow the diffusion of BMP-2 and protect the protein from rapid degradation outside the scaffold. BMP-2 alone in the gel released faster which is the result of a passive diffusion mechanism [59]. We demonstrated that BMP-2 interaction with DS is slightly 10 times weaker than those of BMP-2 with HP. The higher affinity meant that the protein can remain bound for a longer time, exceeding the duration of GAG-protecting mechanism in the ECM [42]. However, our hypothesis is that the interplay between DS and HP with BMP-2 were equally favorable *in vivo*. This proved again the well-known statement that *in vitro* conditions do not necessarily recapitulate the *in vivo* setting. BMP-2 complexed with DS or HP decreases its passive diffusion and retains its activity in the gel. In addition, *in vivo* the cell invasion and degradation of the matrix occurs simultaneously with the passive diffusion mechanism.

Currently it is clear that the wider use of BMP-2 in regenerative medicine will only be possible with the development of a concept that enable for the protein dose reduction in the therapy. In the previous study we reported that the remarkably improved stability of BMP-2 was achieved in acidic pH and glass vial comparing to physiological pH and plastic vial [20]. In another study we showed that BMP-2 stability was enhanced by the presence of HP in the solution [38]. This study established how DS, equally for HP, has a protective capacity towards BMP-2 and uses this stabilization for the incorporation into a gel matrix that provides an extracellular mimetic reservoir to induce bone formation. Since the particular HA gel used in this study and rhBMP-2 (InductOS) has already been used separately in patients, and HP and DS are also already in the clinical use in various forms, the regulatory approval process for human applications maybe accelerated. Although we must be aware that *in vitro* release and even *in vivo* assay in rodents cannot precisely simulate physiological process of human organism. The complexity of human body requires that the final judgment of certain therapy utility cannot be made before clinical trial with long follow up. However, our work highlighted that a broader use of BMP-2 in the clinic is possible and the strategy that allows that is closer to achieve. 

## Materials and Methods

Reported studies using animal subjects were approved (246/8) by the Experimental Animal Committee of Uppsala University and conducted according to the Helsinki guidelines for the use and care of laboratory animals.

### 1: BMP-2 precomplexed with heparin and dermatan sulfate loaded in a HA hydrogel scaffold

Recombinant human bone morphogenetic protein-2 (rhBMP-2; InductOs^®^ former Wyeth Europe) was purchased from Pfizer and reconstituted in the formulation buffer containing 2.5% glycine, 0.5% sucrose, 0.01% polysorbate 80, 5 mM sodium chloride and 5 mM L-glutamic acid at a concentration of 1.5 mg/mL according to the manufacturer’s instructions. GAGs, such as dermatan sulfate (DS; Calbiochem) and heparin (HP; Sigma-Aldrich), both derived from porcine intestinal mucosa, were dissolved at 1 mg per mL of phosphate-buffered saline (PBS, Sigma-Aldrich) and filtered through a sterile 0.22-µm filter. BMP-2 was mixed and preincubated with DS or HP for at least 15 min at room temperature to enable interactions between the growth factor and GAGs. 

The hydrogel components in lyophilized form were purchased from TERMIRA^TM^ (Auxigel; Stockholm, Sweden) and prepared as follows: component A was prepared by dissolving aldehyde-modified hyaluronic acid (HAA) at a concentration of 15 mg/mL in PBS and passing it through a 0.45-µm sterile filter, and component B was prepared by dissolving polyvinyl alcohol with 5% hydrazide functionality (PVAH) at a concentration of 5 mg/mL in deionized water, filtering it through a 0.22-µm sterile filter, and mixing it with BMP-2 that was earlier precomplexed (or not) with DS or HP. The final concentrations of the HAA, PVAH, BMP-2 and GAG components in 1 mL of construct were 9 mg/mL, 1 mg/mL, 25 μg/mL and 125 μg/mL, respectively. According to the manufacturer, the degree of crosslinking (the average percentage of polymer repeat units involved in a crosslink) was 5%. One hour after preparation of the solutions they were combined to form the following constructs: (i) gel/BMP-2+DS, (ii) gel/BMP-2+HP, (iii) gel/BMP-2 and (iiii) gel alone. For the *in vitro* experiments, the construct was formed in the Protein LoBind tubes (Eppendorf) by vigorous mixing by a shaker. For the *in vivo* experiments, components A and B were loaded into two 1-mL sterile Luer-lock syringes (Becton Dickens Medical) that were connected via a Luer-lock adapter (Qosina) and mixed at room temperature 30 times back and forth over a 15 sec period. The gel point was reached after approximately 1 min. 

### 2: In vitro release kinetics of BMP-2 and in vitro bioactivity assessment of released BMP-2

For the *in vitro* release experiment, the samples consisting of 0.2 mL of construct containing 5 μg of BMP-2 and 25 μg of DS or HP, only 5 μg of BMP-2, or hydrogel alone were prepared as described earlier and placed in 1.5-mL Protein LoBind tubes (Eppendorf). The samples were allowed to cure for 3 h at room temperature before being covered with a buffer containing 1 mL of PBS supplemented with 1% bovine serum albumin (BSA, Sigma-Aldrich), 1 mM EDTA and 10 μg/mL heparin (Sigma-Aldrich) (which reduces the degradation of BMP-2) and incubated at room temperature (58). The HA hydrogel without BMP-2 was used as the negative control. In addition, there was a BMP-2 control with DS/HP at a corresponding concentration in PBS solution. Three samples were prepared for each group.

Gel matrices were loaded with BMP-2 and DS or HP; and their release kinetics was evaluated. Release media (200 µl PBS, 1% BSA) from wells containing constructs was collected (50 µl) after the indicated time points and stored in the Protein LoBinding tubes at -20°C. At each time-point the samples were replenished with an equal volume of fresh media (50 µl). On the 30^th^ day, the constructs were treated with a lysis solution containing 100 μL of 500 units/ml hyaluronidase (Sigma-Aldrich) in PBS. The amount of BMP-2 in each sample was determined by a sandwich enzyme linked immunosorbent assay (ELISA) kit (Human BMP-2 Quantikine; R&D Systems) as per the manufacturer's instructions. BMP-2 cumulative release was expressed as a percentage of the starting concentration. The absorbance of the samples was read at 450 nm and normalized at 570 nm using the Kinetic Microplate Reader (Molecular Devices). The blank release media was used as a background correction for BMP-2 levels; and this value at each time point 15 min, 30 min, 1h, 3h, 6h, 16h, 24h, 2 days, 7 days, 14 days, 21 days and 30 days) was subtracted from the value obtained from the release media containing gel/BMP-2with or without DS or HP.

The bioactivity of BMP-2 released from the constructs *in vitro* was determined by its capacity to stimulate the alkaline phosphatase (ALP) activity of the mouse myoblast cell line C2C12 purchased from the American Type Culture Collection (ATCC–LGC Standards, Sweden). C2C12 cells were employed because there is a confirmed correlation between the ALP activity and amount of bioactive BMP-2 in this particular cell line [3,9]. The cells were cultured in Dulbecco’s modified Eagle’s medium (DMEM, Gibco) supplemented with 10% (v/v) fetal bovine serum (Gibco) and 100 U ml^-1^ penicillin and streptomycin (Gibco) at 37°C in a 5% CO_2_-95% air atmosphere. The cells were seeded at 5x10^3^/mL in 96-well plates in the culture media. After 24 h, the culture media was changed to media containing 10 vol% of release media that was obtained in the *in vitro* release experiment. After 48 h, the cells were washed with PBS and lysed by two freeze-thaw cycles at -80°C and 37°C (30 and 20 min for the first and second cycles, respectively). The ALP activity in this extracted solution was determined using the p-nitrophenol (pNP) concentration obtained from 100 µL of substrate (p-nitrophenyl phosphate; Sigma-Aldrich) in 0.1 M glycine, 1 mM MgCl_2_, and 1 mM ZnCl_2_ at a pH of 10.4 at 37°C. After 20 min, 50 µL of 0.1 M NaOH was added to stop the reaction. The absorbance was read at 405 nm, which corresponds to pNP concentrations determined using the Kinetic Microplate Reader (Molecular Devices). Each ALP activity measurement was normalized to the total sample protein content quantified using the NanoDrop (ND-1000, Thermo Scientific). The experiments were performed in triplicate. 

### 3: Kinetic interactions between BMP-2 and DS/HP

To determine the binding affinity and kinetics between BMP-2 and DS or HP, the interaction was measured using Biacore™ X100 and SA sensor chips that are pre-coated with streptavidin (GE Healthcare). A solution of 20 mM phosphate-buffered saline, 2.7 mM KCl, 137 mM NaCl, and 0.05% surfactant P20 (pH 7.4) was prepared from a stock solution (10X PBS-P+, GE Healthcare) and used as running buffer. After docking the SA sensor chip and priming the instrument with running buffer, the chip was preconditioned with three injections (1 min each) of 40 mM NaOH (GE Healthcare) and 1 M NaCl (Merck) (NaOH/NaCl) following the recommendations provided by the Biacore X100 software. Biotinylated HP from porcine intestinal mucosa (Sigma-Aldrich) and biotinylated DS from porcine intestinal mucosa were prepared as described elsewhere [36] (gift from Silesia University) and dissolved in 10 mM sodium acetate (pH 5.5). DS or HP was immobilized by injecting approximately 1 µg/mL of solution into flow cell 2. Flow cell 1 was used for the reference subtraction of all signals. Immobilization levels were kept relatively low, below 20 RU, to minimize the possibility that BMP-2 binds to two HP or DS molecules simultaneously. In each binding cycle, BMP-2 was injected for 150 s, followed by the monitoring of dissociation for 300 s while running buffer was flowing over the surface at a flow rate of 30 μL/min. Each association/dissociation step was followed by 30 s of regeneration with NaOH/NaCl and a 1-min stabilization period before the next binding cycle started. An initial series of 3 buffer injections was performed to equilibrate the system. BMP-2 binding was monitored using a three-fold dilution series with six concentrations ranging from 0.2 to 50 nM. Before, during and after the dilution series, buffer solutions were injected, and the average of these three “blank” cycles was used for a second reference subtraction of all binding data. For the kinetic analyses, data were globally fit to a 1:1 interaction model to obtain binding parameters. Analysis of the binding kinetics was performed using Biacore™ T200 evaluation software (GE Healthcare). The apparent equilibrium dissociation constant (Kd) was calculated as the ratio of k_d_/k_a_. 

### 4: Efficacy of in vivo bone formation

#### 4.1: Study design

Six adult male Sprague-Dawley rats (Taconic M&B, Lille Skensved, Denmark), aged 9-10 weeks, weighing approximately 280-300 g, were anesthetized using isoflurane (4,5% induction, 2.5% maintenance; Forene®, Abbott Scandinavia), starting with flow rates of 4 L/min oxygen and 4 L /min in isoflurane in an induction chamber and then by mask at flow rates of 1.5 L/min oxygen, 1.5 L/min air and 3 /min isoflurane. After shaving, the lumbar region was disinfected with three washes of 70% ethanol. Each construct of 0.2 mL in volume (n=6) was injected subcutaneously with a 21-G needle at a minimum depth of 15 mm. The HA hydrogels did or did not contain 4 µg of BMP-2 and 20 μg of DS or HP per injection. The HA hydrogel without BMP-2 or GAGs was considered a negative control. Immediately after the intervention, animals received 0.05 mg/kg buprenorphine (Temgesic, Schering-Plough) subcutaneously, after which time the animals had no signs of pain or distress. The animals were housed with two rats per cage with 12/12 h light/dark cycles in a controlled environment (temperature 22°C and humidity 45 ± 10%) with *ad libitum* access to food and water. Six weeks after the injection, the rats were sacrificed via CO_2_ asphyxiation, and ectopic ossification which occurred in the upper region of the back were harvested. The bone samples were fixed in 4% paraformaldehyde in PBS, which was replaced with 70% ethanol after 24 h at room temperature. 

#### 4.2: Determination of bone volume

The ectopic bone formation was evaluated by micro-CT using a Skyscan 1072 system (Kontich) with 100 kV/98 µA X-ray source. For quantitative 3D analysis, the specimens were placed vertically onto the sample holder of the micro-CT imaging system. Subsequently, a high-resolution scan was performed at a final resolution of 14.53-μm (20x magnification; 3.9-s exposure time; 1-mm filter applied). Then, using Nrecon V1.4 (SkyScan), a cone beam reconstruction was performed on the projected files. The bone volume-to-total tissue volume (BV/TV) ratio, trabecular thickness (Tb. Th) and trabecular separation (Tb. Sp.) of the ectopic bone samples was calculated by morphometric analysis with segmentation thresholds of 41-159 (CT analyzer; software version 1.10.1.0, SkyScan). Data from each group are expressed as mean ± standard deviation (n=6). Finally, using a 3D creator software (3D-DOCTOR 4.0, Able Software Corp.), 3D reconstructions of the samples were also obtained. 

#### 4.3: Bone structure assessment using histology

The specimens stored in 70% ethanol were further processed for evaluation by histology. The ectopic bones were completely decalcified using an electrophoresis system (Tissue-Tek Miles scientific, Histolab) with formic acid, dehydrated, and embedded in paraffin wax. Serial cross sections were cut from the middle part of the specimens with a microtome at a thickness of 5 μm, deparaffinized and stained using Masson’s Trichrome (Merck). The histological sections were photographed using a bright-field microscope (Eclipse TE 2000U, Nikon). 

#### 4.4: Bone structure assessment using electron microscopy

For the SEM analysis, undecalcified samples were embedded in poly(methylmethacrylate) [21]. Briefly, the samples were immersed in methylmethacrylate (MMA) for 24 h at 4°C. Then, they were incubated in MMA with 2% benzoyl peroxide (BP) for 3 days at 4°C. Finally, they were placed in a solution of 100 mL of MMA containing 4 g of BP and 25 mL of dibutylphthalate and stored in sealed glass vials at 4°C for 7 days and then 14 days at room temperature. The sample blocks were polished through a series of silicon carbide paper ranging from P220 to P2400 (Struers), corresponding to a grain size range of 60 to 5 µm. Once the surfaces were sufficiently even and smooth, they were double-coated with gold and cemented on SEM stud supports using carbon cement (Struers). SEM of the samples was performed with an INSPECT F (FEI,England) instrument at 10 keV (magnifications and other details are provided in the images themselves). Using the INCA software, images were further analyzed by elemental mapping of the sample surfaces at 10 keV with an objective aperture of 3 and spot size of 5.0. The data were collected at a rate of 14 frames per sample using secondary electrons detection.

#### 4.5: Evaluation of osteogenic and angiogenic markers

Paraffin sections were immunostained with primary antibodies specific for rat osteocalcin (OC; Santa Cruz Biotechnology), rat CD31 (BD) and rat alfa-smoth muscle actin (α-SMA; Dako). The sections were placed in a Tris/EDTA solution (pH 9; Dako), incubated at 95°C for 20 min, washed with PBS and blocked in a 12% BSA solution. Slides were then labeled with the primary antibodies, namely OC (1:50), CD31 (1:50), and α-SMA (1:100), and diluted in PBS containing 12% bovine serum albumin (BSA). After overnight incubation at 4°C, the sections were washed three times for 5 min in PBS and blocked for additional 15 min. To visualize the primary antibody, TRITC- or FITC-conjugated polyclonal goat F(ab’)_2_ fragments directed at mouse or rabbit immunoglobulins (Dako) were added to the sections. Slides were washed three times for 5 min in PBS. Some sections of cranial bone (positive control) and some sections not treated with the primary antibody (negative control) were used. Finally, the slides were incubated for 5 min in PBS containing 1 µg/ml Hoechst 33342 (Sigma), rewashed with PBS, and mounted with Dako mounting solution (Dako). Images were taken with a DXM1200F digital camera connected to a Nikon Eclipse TE2000-U inverted microscope. The device is equipped with Hoechst 33342, FITC, and TRITC filter sets (Nikon AG, Switzerland; Software: Nikon ACT-1 vers. 2.70). Images were processed with Photoshop 7.0 (Adobe Systems Inc.).

### 5: Statistical analysis

Statistical analysis was performed with PASW Statistics 16.0 (SPSS Inc.). Student’s t-test was used to analyze the significant differences between all groups based on the consideration that a p-value less than 0.05 was significant.

## Conclusions

In our study, first we investigated the ability of DS and HP to protect BMP-2 and control its release from a hydrogel scaffold *in vitro*. The release study showed that BMP-2 precomplexed with DS or HP provided prolonged protein release compared with the same system with non complexed BMP-2. Although HP showed a slightly higher affinity to BMP-2 than DS, both constructs yielded equal volumes of ectopic bone formation. The amount of bone formed with precomplexed BMP-2 was two times larger and exhibited more abundant trabecular network compared with the HA hydrogel with BMP-2 alone. Our findings further highlighted that molecular interactions occurring between BMP-2 and DS are as important as those between BMP-2 and HP, as well as that by modulating the protection and delivery of the growth factor, significant enhancements in bone formation can be achieved. This knowledge can accelerate development of alternative approaches for safer bone fracture treatment. Presented strategy uses starting materials and components that are in clinical use today. This strategy may lead to decrease required dose of BMP-2 reducing the risks of side effects and therapy costs. 

## Supporting Information

Figure S1 **BMP-2, either alone or precomplexed with dermatan sulfate (DS) or heparin (HP), was added to polyvinyl alcohol (PVAH) and hyaluronic acid aldehyde (HAA).** All components were mixed to form a gel construct.
(TIF)

Figure S2 **As a control, the cross sections of rat cranium was immunostained with osteocalcin (OC, red), CD31 (red) and α-SMA (green).** The erythrocytes were visualized by green autofluorescence. Cell nuclei were stained with DAPI (blue). The images are shown merged.
(TIF)

Figure S3 **A schematic representation showing the mechanism of the BMP-2 release process.** The low rate of diffusion of BMP-2 protected by complexation using dermatan sulfate or heparin (gel/BMP-2+DS or HP) in comparison with the higher rate of diffusion of non-complexed BMP-2 (gel/BMP-2).
(TIF)

## References

[1] Heiple KG, Chase SW, Herndon CH (1963) A Comparative Study of the Healing Process Following Different Types of Bone Transplantation. J Bone Joint Surg Am 45: 1593-1616. PubMed: 14083136. 14083136

[2] Younger EM, Chapman MW (1989) Morbidity at bone graft donor sites. J Orthop Trauma 3: 192-195. doi:10.1097/00005131-198909000-00002. PubMed: 2809818. 2809818

[3] Cheng H, Jiang W, Phillips FM, Haydon RC, Peng Y et al. (2003) Osteogenic activity of the fourteen types of human bone morphogenetic proteins (BMPs). J Bone Joint Surg Am 85-A: 1544-1552. PubMed: 12925636. 12925636 10.2106/00004623-200308000-00017

[4] Okubo Y, Bessho K, Fujimura K, Iizuka T, Miyatake SI (2000) Osteoinduction by bone morphogenetic protein-2 via adenoviral vector under transient immunosuppression. Biochem Biophys Res Commun 267: 382-387. doi:10.1006/bbrc.1999.1975. PubMed: 10623628. 10623628

[5] Kusumoto K, Bessho K, Fujimura K, Akioka J, Ogawa Y et al. (1998) Prefabricated muscle flap including bone induced by recombinant human bone morphogenetic protein-2: an experimental study of ectopic osteoinduction in a rat latissimus dorsi muscle flap. Br J Plast Surg 51: 275-280. doi:10.1054/bjps.1998.0008. PubMed: 9771344. 9771344

[6] Uludag H, D'Augusta D, Golden J, Li J, Timony G et al. (2000) Implantation of recombinant human bone morphogenetic proteins with biomaterial carriers: A correlation between protein pharmacokinetics and osteoinduction in the rat ectopic model. J Biomed Mater Res 50: 227-238. doi:10.1002/(SICI)1097-4636(200005)50:2. PubMed: 10679688. 10679688

[7] Wozney JM, Rosen V, Celeste AJ, Mitsock LM, Whitters MJ et al. (1988) Novel regulators of bone formation: molecular clones and activities. Science 242: 1528-1534. doi:10.1126/science.3201241. PubMed: 3201241. 3201241

[8] Caetano-Lopes J, Lopes A, Rodrigues A, Fernandes D, Perpétuo IP et al. (2011) Upregulation of Inflammatory Genes and Downregulation of Sclerostin Gene Expression Are Key Elements in the Early Phase of Fragility Fracture Healing. PLOS ONE 6: e16947 PubMed: 21347301. 21347301 10.1371/journal.pone.0016947 PMC3037947

[9] Zhao B, Katagiri T, Toyoda H, Takada T, Yanai T et al. (2006) Heparin potentiates the in vivo ectopic bone formation induced by bone morphogenetic protein-2. J Biol Chem 281: 23246-23253. doi:10.1074/jbc.M511039200. PubMed: 16754660. 16754660

[10] Govender S, Vadasz P (2002) Weak non-linear analysis of moderate Stefan number stationary convection in rotating mushy layers. Transp Porous Media 49: 247-263. doi:10.1023/A:1016241225343.

[11] Berner A, Reichert JC, Müller MB, Zellner J, Pfeifer C et al. (2012) Treatment of long bone defects and non-unions: from research to clinical practice. Cell Tissue Res 347: 501-519. doi:10.1007/s00441-011-1184-8. PubMed: 21574059. 21574059

[12] Shields LBE, Raque GH, Glassman SD, Campbell M, Vitaz T et al. (2006) Adverse effects associated with high-dose recombinant human bone morphogenetic protein-2 use in anterior cervical spine fusion. Spine 31: 542-547. doi:10.1097/01.brs.0000201424.27509.72. PubMed: 16508549. 16508549

[13] Neovius E, Lemberger M, Docherty Skogh AC, Hilborn J, Engstrand T (2012) Alveolar bone healing accompanied by severe swelling in cleft children treated with bone morphogenetic protein-2 delivered by hydrogel. J Plast Reconstr Aesthet Surg, 66: 37–42. PubMed: 22980542. 22980542 10.1016/j.bjps.2012.08.015

[14] Friess W, Uludag H, Foskett S, Biron R, Sargeant C (1999) Characterization of absorbable collagen sponges as rhBMP-2 carriers. Int J Pharm 187: 91-99. doi:10.1016/S0378-5173(99)00174-X. PubMed: 10502616. 10502616

[15] Carragee EJ, Hurwitz EL, Weiner BK. (2011) A critical review of recombinant human bone morphogenetic protein-2 trials in spinal surgery: emerging safety concerns and lessons learned. Spine J 11: 471-491. doi:10.1016/j.spinee.2011.04.023. PubMed: 21729796. 21729796

[16] Fu YC, Nie H, Ho ML, Wang CK, Wang CH (2008) Optimized bone regeneration based on sustained release from three-dimensional fibrous PLGA/HAp composite scaffolds loaded with BMP-2. Biotechnol Bioeng 99: 996-1006. doi:10.1002/bit.21648. PubMed: 17879301. 17879301

[17] Sawyer AA, Song SJ, Susanto E, Chuan P, Lam CX et al. (2009) The stimulation of healing within a rat calvarial defect by mPCL-TCP/collagen scaffolds loaded with rhBMP-2. Biomaterials 30: 2479-2488. doi:10.1016/j.biomaterials.2008.12.055. PubMed: 19162318. 19162318

[18] Jeon O, Song SJ, Yang HS, Bhang SH, Kang SW et al. (2008) Long-term delivery enhances in vivo osteogenic efficacy of bone morphogenetic protein-2 compared to short-term delivery. Biochem Biophys Res Commun 369: 774-780. doi:10.1016/j.bbrc.2008.02.099. PubMed: 18313401. 18313401

[19] Yang HS, La WG, Bhang SH, Jeon JY, Lee JH et al. (2010) Heparin-Conjugated Fibrin as an Injectable System for Sustained Delivery of Bone Morphogenetic Protein-2. Tissue Eng A 16: 1225-1233. doi:10.1089/ten.tea.2009.0390. PubMed: 19886733. 19886733

[20] Kisiel M, Ventura M, Oommen OP, George A, Walboomers XF et al. (2012) Critical assessment of rhBMP-2 mediated bone induction: An in vitro and in vivo evaluation. J Control Release 162: 646-653. doi:10.1016/j.jconrel.2012.08.004. PubMed: 22902595. 22902595

[21] Martínez-Sanz E, Varghese OP, Kisiel M, Engstrand T, Reich KM et al. (2012) Minimally invasive mandibular bone augmentation using injectable hydrogels. J Tissue Eng Regen Med, 6 Suppl 3: s15–23. PubMed: 22941759. 22941759 10.1002/term.1593

[22] Patterson J, Siew R, Herring SW, Lin AS, Guldberg R et al. (2010) Hyaluronic acid hydrogels with controlled degradation properties for oriented bone regeneration. Biomaterials 31: 6772-6781. doi:10.1016/j.biomaterials.2010.05.047. PubMed: 20573393. 20573393 PMC2907529

[23] Kisiel M, Klar AS, Martino MM, Ventura M, Hilborn J (2013) Evaluation of injectable constructs for bone repair with a subperiosteal cranial model in the rat. PLOS ONE 8: e71683. doi:10.1371/journal.pone.0071683. PubMed: 23967235. 23967235 PMC3742484

[24] Toole BP (2004) Hyaluronan: from extracellular glue to pericellular cue. Nat Rev Cancer 4: 528-539. doi:10.1038/nrc1391. PubMed: 15229478. 15229478

[25] Schiller J, Fuchs B, Arnhold J, Arnold K (2003) Contribution of reactive oxygen species to cartilage degradation in rheumatic diseases: molecular pathways, diagnosis and potential therapeutic strategies. Curr Med Chem 10: 2123-2145. doi:10.2174/0929867033456828. PubMed: 12871089. 12871089

[26] Csoka AB, Frost GI, Stern R (2001) The six hyaluronidase-like genes in the human and mouse genomes. Matrix Biol 20: 499-508. doi:10.1016/S0945-053X(01)00172-X. PubMed: 11731267. 11731267

[27] Lepperdinger G, Müllegger J, Kreil G (2001) Hyal2--less active, but more versatile? Matrix Biol 20: 509-514. doi:10.1016/S0945-053X(01)00170-6. PubMed: 11731268. 11731268

[28] Bergman K, Engstrand T, Hilborn J, Ossipov D, Piskounova S et al. (2009) Injectable cell-free template for bone-tissue formation. J Biomed Mater Res A 91A: 1111-1118. doi:10.1002/jbm.a.32289. PubMed: 19107794. 19107794

[29] Kisiel M, Martino MM, Ventura M, Hubbell JA, Hilborn J et al. (2012) Improving the osteogenic potential of BMP-2 with hyaluronic acid hydrogel modified with integrin-specific fibronectin fragment. Biomaterials, 34: 704–12. PubMed: 23103154. 23103154 10.1016/j.biomaterials.2012.10.015

[30] Nageeb M, Nouh SR, Bergman K, Nagy NB, Khamis D et al. (2012) Bone Engineering by Biomimetic Injectable Hydrogel. Mol Cryst Liq Cryst 555: 177-188. doi:10.1080/15421406.2012.635530.

[31] Liu HW, Chen CH, Tsai CL, Lin IH, Hsiue GH (2007) Heterobifunctional poly(ethylene glycol)-tethered bone morphogenetic protein-2-stimulated bone marrow mesenchymal stromal cell differentiation and osteogenesis. Tissue Eng 13: 1113-1124. doi:10.1089/ten.2006.0209. PubMed: 17355208. 17355208

[32] Jha AK, Yang W, Kirn-Safran CB, Farach-Carson MC, Jia X (2009) Perlecan domain I-conjugated, hyaluronic acid-based hydrogel particles for enhanced chondrogenic differentiation via BMP-2 release. Biomaterials 30: 6964-6975. doi:10.1016/j.biomaterials.2009.09.009. PubMed: 19775743. 19775743 PMC2783995

[33] Jeon O, Song SJ, Kang SW, Putnam AJ, Kim BS (2007) Enhancement of ectopic bone formation by bone morphogenetic protein-2 released from a heparin-conjugated poly(L-lactic-co-glycolic acid) scaffold. Biomaterials 28: 2763-2771. doi:10.1016/j.biomaterials.2007.02.023. PubMed: 17350678. 17350678

[34] Takada T, Katagiri T, Ifuku M, Morimura N, Kobayashi M et al. (2003) Sulfated polysaccharides enhance the biological activities of bone morphogenetic proteins. J Biol Chem 278: 43229-43235. doi:10.1074/jbc.M300937200. PubMed: 12912996. 12912996

[35] Lyon M, Deakin JA, Rahmoune H, Fernig DG, Nakamura T et al. (1998) Hepatocyte growth factor scatter factor binds with high affinity to dermatan sulfate. J Biol Chem 273: 271-278. doi:10.1074/jbc.273.1.271. PubMed: 9417075. 9417075

[36] Koźma EM, Wisowski G, Olczyk K (2009) Platelet derived growth factor BB is a ligand for dermatan sulfate chain(s) of small matrix proteoglycans from normal and fibrosis affected fascia. Biochimie 91: 1394-1404. doi:10.1016/j.biochi.2009.07.010. PubMed: 19631712. 19631712

[37] Johnson MR, Boerckel JD, Dupont KM, Guldberg RE (2011) Functional restoration of critically sized segmental defects with bone morphogenetic protein-2 and heparin treatment. Clin Orthop Relat Res 469: 3111-3117. doi:10.1007/s11999-011-2012-x. PubMed: 21863396. 21863396 PMC3183200

[38] Bramono DS, Murali S, Rai B, Ling L, Poh WT et al. (2012) Bone marrow-derived heparan sulfate potentiates the osteogenic activity of bone morphogenetic protein-2 (BMP-2). Bone 50: 954-964. doi:10.1016/j.bone.2011.12.013. PubMed: 22227436. 22227436 PMC3589980

[39] Piskounova S, Gedda L, Hulsart-Billström G, Hilborn J, Bowden T (2012) Characterization of recombinant human bone morphogenetic protein-2 delivery from injectable hyaluronan-based hydrogels by means of (125) I-radiolabelling. J Tissue Eng Regen Med. PubMed: 22927307 10.1002/term.1584 22927307

[40] Santos MI, Reis RL (2010) Vascularization in bone tissue engineering: physiology, current strategies, major hurdles and future challenges. Macromol Biosci 10: 12-27. doi:10.1002/mabi.200900107. PubMed: 19688722. 19688722

[41] Traktuev DO, Prater DN, Merfeld-Clauss S, Sanjeevaiah AR, Saadatzadeh MR et al. (2009) Robust Functional Vascular Network Formation in Vivo by Cooperation of Adipose Progenitor and Endothelial Cells. Circ Res 104: 1410-U1320. doi:10.1161/CIRCRESAHA.108.190926. PubMed: 19443841. 19443841

[42] Ruppert R, Hoffmann E, Sebald W (1996) Human bone morphogenetic protein 2 contains a heparin-binding site which modifies its biological activity. Eur J Biochem 237: 295-302. doi:10.1111/j.1432-1033.1996.0295n.x. PubMed: 8620887. 8620887

[43] Massagué J (1990) The transforming growth factor-beta family. Annu Rev Cell Biol 6: 597-641. doi:10.1146/annurev.cb.06.110190.003121. PubMed: 2177343. 2177343

[44] Xu X, Jha AK, Duncan RL, Jia X (2011) Heparin-decorated, hyaluronic acid-based hydrogel particles for the controlled release of bone morphogenetic protein 2. Acta Biomaterialia 7: 3050-3059. doi:10.1016/j.actbio.2011.04.018. PubMed: 21550426. 21550426 PMC3188452

[45] Jiao X, Billings PC, O'Connell MP, Kaplan FS, Shore EM et al. (2007) Heparan sulfate proteoglycans (HSPGs) modulate BMP2 osteogenic bioactivity in C2C12 cells. J Biol Chem 282: 1080-1086. PubMed: 17020882. 17020882 10.1074/jbc.M513414200

[46] Bhakta G, Rai B, Lim ZXH, Hui JH, Stein GS et al. (2012) Hyaluronic acid-based hydrogels functionalized with heparin that support controlled release of bioactive BMP-2. Biomaterials 33: 6113-6122. doi:10.1016/j.biomaterials.2012.05.030. PubMed: 22687758. 22687758 PMC3628623

[47] Jones G, Sambrook PN (1994) Drug-induced disorders of bone metabolism. Incidence, management and avoidance. Drug Saf 10: 480-489. doi:10.2165/00002018-199410060-00006. PubMed: 7917076. 7917076

[48] Wolinsky-Friedland M (1995) Drug-induced metabolic bone disease. Endocrinol Metab Clin North Am 24: 395-420. PubMed: 7656896. 7656896

[49] Manton KJ, Leong DF, Cool SM, Nurcombe V (2007) Disruption of heparan and chondroitin sulfate signaling enhances mesenchymal stem cell-derived osteogenic differentiation via bone morphogenetic protein signaling pathways. Stem Cells 25: 2845-2854. doi:10.1634/stemcells.2007-0065. PubMed: 17702986. 17702986

[50] Trowbridge JM, Rudisill JA, Ron D, Gallo RL (2002) Dermatan sulfate binds and potentiates activity of keratinocyte growth factor (FGF-7). J Biol Chem 277: 42815-42820. doi:10.1074/jbc.M204959200. PubMed: 12215437. 12215437

[51] Osses N, Gutierrez J, Lopez-Rovira T, Ventura F, Brandan E (2006) Sulfation is required for bone morphogenetic protein 2-dependent Id1 induction. Biochem Biophys Res Commun 344: 1207-1215. doi:10.1016/j.bbrc.2006.04.029. PubMed: 16647687. 16647687

[52] Radek KA, Taylor KR, Gallo RL (2009) FGF-10 and specific structural elements of dermatan sulfate size and sulfation promote maximal keratinocyte migration and cellular proliferation. Wound Repair Regen 17: 118-126. doi:10.1111/j.1524-475X.2008.00449.x. PubMed: 19152659. 19152659 PMC2721336

[53] Ornitz DM, Herr AB, Nilsson M, Westman J, Svahn CM et al. (1995) FGF binding and FGF receptor activation by synthetic heparan-derived di- and trisaccharides. Science 268: 432-436. doi:10.1126/science.7536345. PubMed: 7536345. 7536345

[54] Barger-Lux MJ, Recker RR (2002) Bone microstructure in osteoporosis: transilial biopsy and histomorphometry. Top Magn Reson Imaging 13: 297-305. doi:10.1097/00002142-200210000-00002. PubMed: 12464743. 12464743

[55] Peacock M (2010) Calcium metabolism in health and disease. Clin J Am Soc Nephrol 5 Suppl 1: S23-S30. doi:10.2215/CJN.05910809. PubMed: 20089499. 20089499

[56] Afonso L, Bandaru H, Rathod A, Badheka A, Ali Kizilbash M et al. (2011) Prevalence, determinants, and clinical significance of cardiac troponin-I elevation in individuals admitted for a hypertensive emergency. J Clin Hypertens (Greenwich) 13: 551-556. doi:10.1111/j.1751-7176.2011.00476.x. PubMed: 21806764. 21806764 PMC8108865

[57] Chao EYS, Inoue N, Elias JJ, Aro H (1998) Enhancement of fracture healing by mechanical and surgical intervention. Clin Orthop Relat Res: S163-S178. PubMed: 9917637. 9917637 10.1097/00003086-199810001-00018

[58] Clarke B (2008) Normal Bone Anatomy and Physiology. Clin J Am Soc Nephrol 3: S131-S139. doi:10.2215/CJN.04151206. PubMed: 18988698. 18988698 PMC3152283

[59] Laub M, Seul T, Schmachtenberg E, Jennissen HP (2001) Molecular modelling of bone morphogenetic protein-2 (BMP-2) by 3D-rapid prototyping. Materialwiss Werkstofftech 32: 926-930. doi:10.1002/1521-4052(200112)32:12.

