# Supplementary figures and images for: Complexation and Sequestration of BMP-2 from an ECM Mimetic Hyaluronan Gel for Improved Bone Formation
Source: PLoS One. 2013 Oct 22;8(10):e78551. doi: 10.1371/journal.pone.0078551 (PMC3805527; doi:10.1371/journal.pone.0078551)

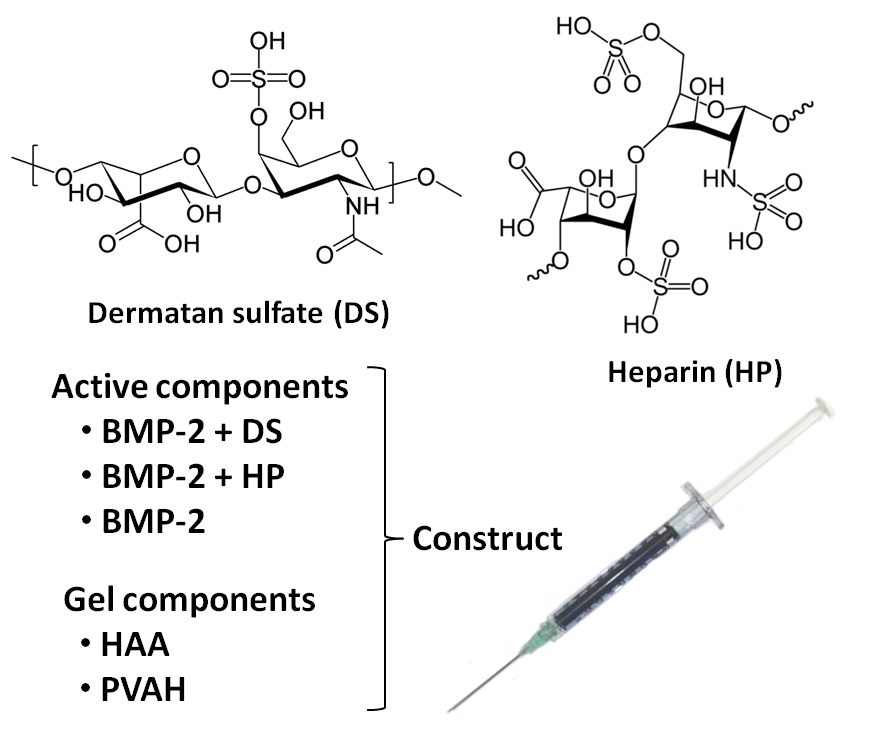

Supplement: Figure S1 — BMP-2, either alone or precomplexed with dermatan sulfate (DS) or heparin (HP), was added to polyvinyl alcohol (PVAH) and hyaluronic acid aldehyde (HAA). All components were mixed to form a gel construct. (TIF) [file pone.0078551.s001.tif]

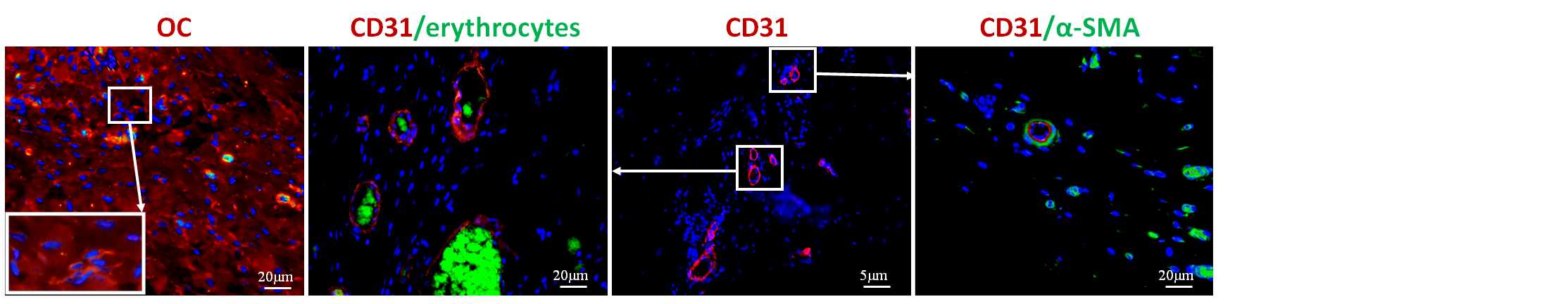

Supplement: Figure S2 — As a control, the cross sections of rat cranium was immunostained with osteocalcin (OC, red), CD31 (red) and α-SMA (green). The erythrocytes were visualized by green autofluorescence. Cell nuclei were stained with DAPI (blue). The images are shown merged. (TIF) [file pone.0078551.s002.tif]

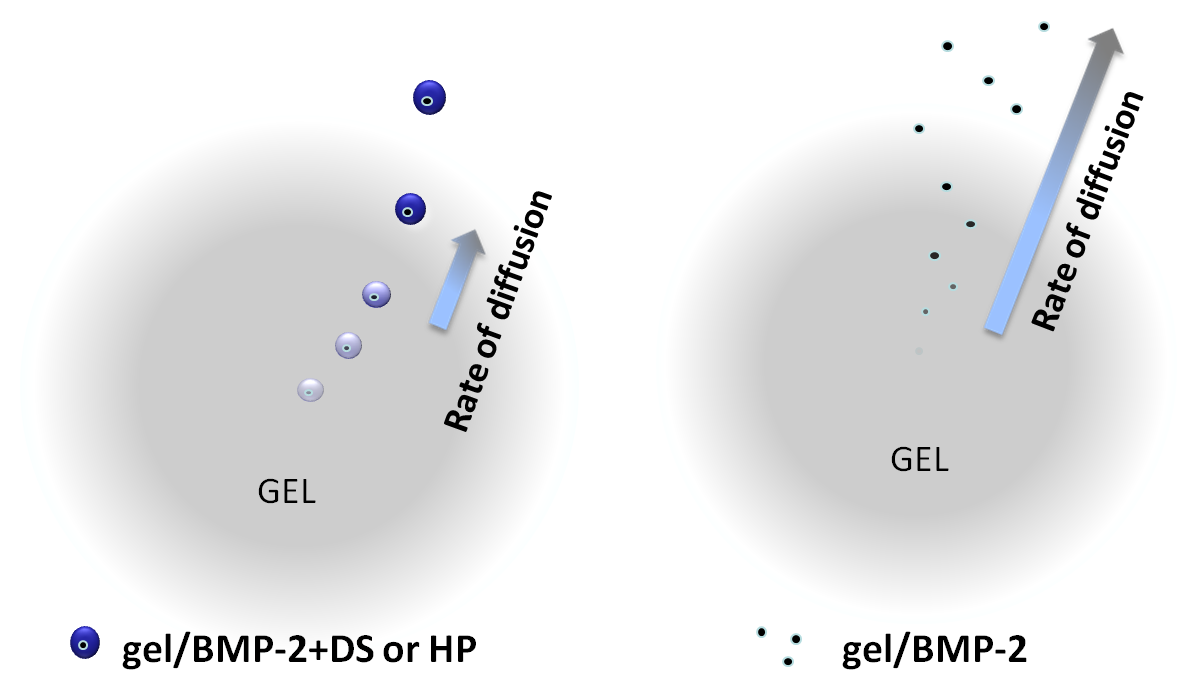

Supplement: Figure S3 — A schematic representation showing the mechanism of the BMP-2 release process. The low rate of diffusion of BMP-2 protected by complexation using dermatan sulfate or heparin (gel/BMP-2+DS or HP) in comparison with the higher rate of diffusion of non-complexed BMP-2 (gel/BMP-2). (TIF) [file pone.0078551.s003.tif]
